# Comparing Outcomes in Patients Undergoing Coronary Artery Bypass Grafting With and Without Using the Internal Mammary Artery in a Tertiary Care Hospital

**DOI:** 10.7759/cureus.10427

**Published:** 2020-09-13

**Authors:** Yasir Khan, Syed Shahabuddin, Shiraz Hashmi, Shahid Sami

**Affiliations:** 1 Cardiothoracic Surgery, Aga Khan University Hospital, Karachi, PAK; 2 Cardiothoracic Surgery, Aga Khan University, Karachi, PAK

**Keywords:** left internal mammary artery, developing country

## Abstract

Purpose

The left internal mammary artery in coronary artery bypass grafting (CABG) is considered to be an important intraoperative quality indicator with excellent long-term results. The purpose of this study is to compare the early outcomes of CABG with and without the left internal mammary artery (LIMA) in the South Asian population and to look for the utilization of LIMA as per the recommendations of the Society of Thoracic Surgeons in a developing country.

Materials and methods

A retrospective review was carried out for all patients who underwent CABG from 2010 to 2015. Patients were divided into two groups on the basis of whether LIMA was used (Group I) or not used (Group II) as a conduit. Both groups were further subdivided into elective and urgent. Preoperative, intraoperative, and postoperative variables were recorded and compared.

Results

After exclusion, a total of 2619 patients underwent isolated CABG surgery during the required duration. The LIMA was used in 94% of the patients (n=2472) while 147 patients underwent CABG without LIMA. The use of LIMA was associated with significantly lower mortality (2% LIMA vs 8.8% no LIMA), as well as a decrease in major comorbidity, stroke, and prolonged ventilation. In the subgroup analysis, LIMA usage in elective and urgent cases was associated with significantly lower mortality (elective 1.6% LIMA vs 7.4% No LIMA) (urgent 4.8% LIMA vs 15.7 % no LIMA)) and major morbidity.

Conclusion

The outcomes of CABG procedures without LIMA were not encouraging. Our results support compliance with standard adult cardiac surgery quality-of-care guidelines.

## Introduction

Coronary artery bypass grafting (CABG) with arterial and vein graft conduits is the most commonly performed cardiac surgical procedure for coronary artery disease worldwide. Arterial grafts are considered superior in terms of patency and durability. The most important and commonly used arterial graft is the left internal mammary artery (LIMA) due to its increased patency rate and resistance to atherosclerosis [1]. It is widely accepted that using LIMA as a conduit for the left anterior descending (LAD) artery is associated with better long-term survival than using the long saphenous vein [2]. Apart from the clinical importance, the intraoperative usage of at least one (right or left) internal mammary artery (IMA) in CABG reflects compliance with quality assurance, as it is also considered an important intraoperative quality indicator [3]. However, its use is precluded at times in certain subsets of patients, including the elderly, very obese, those who have received mediastinal radiation, the emergent nature of the procedure, and the presence of composite risk factors [4]. Although the long-term benefits of using LIMA to LAD are well-established and superior to vein grafts, the short-term outcomes may not have a significant impact [5]. The majority of the studies comparing the left internal mammary artery with the saphenous vein graft comes from the western population. Few studies have been performed in South Asia comparing these two groups [6]. For a quality cardiac center, the Society of Thoracic Surgeons (STS) recommends the usage of 90%-95% LIMA for CABG [7]. This study is performed to evaluate the short-term outcome in terms of morbidity and mortality as defined by STS in adult patients undergoing CABG with and without using the internal mammary artery at a tertiary care hospital in the south Asian population. The secondary outcome was to compare the quality of care given at a tertiary hospital in a developing country as per the recommendation of international adult cardiac surgery quality of care.

## Materials and methods

The patients undergoing a first-time CABG from January 2010 to December 2016 at our hospital were retrospectively reviewed in this study. The data were collected prospectively in the cardiothoracic surgery sectional computerized database using a standardized data set and definitions. The institutional ethical review committee (ERC) granted an exemption to carry out this project. A total of 2864 patients were included in this study. This analysis excluded patients who had previous CABG and those patients who underwent an emergent and salvage procedure. In addition, patients who underwent concomitant valve procedures were excluded from the study. After applying this exclusion, a total of 2619 patients was left for analysis.

Study variables

Our database has accurate patient information consisting of patient demographics, preoperative risk factors, operative information, and short-term postoperative outcomes, including morbidity and mortality. This database is updated on a regular basis.

In addition to age and gender, the following variables were assessed: diabetes mellitus, chronic obstructive pulmonary disease, cerebrovascular disease, congestive heart failure New York Heart Association (NYHA) class, myocardial infarction. Information immediately preceding surgery was also obtained, which included preoperative creatinine level, most recent ejection fraction, and details about coronary artery disease that included the number of diseased vessels. Information regarding the use of an intra-aortic balloon pump was also obtained.

Variables describing the procedure itself were also collected, which included surgical priority defined as urgent, emergent, salvage, elective, and graft types, including the saphenous vein and internal mammary artery. The duration of the procedure in terms of cross-clamp time and pump time was obtained.

This study examined multiple outcomes that were also reported in our database. These outcomes include early mortality and morbidity like prolonged ventilation, reoperation for bleeding, stroke, surgical site infection, and length of stay.

All this information was collected on a set proforma. The outcomes were defined as per STS:

Mortality: Defined as death during the hospital stay or within 30 days after discharge

Reopening: Patients who require a return to the operating room reopening for any cause

Stroke: Defined as a new central neurological deficit persisting for more than 24 hours

Deep sternal wound infection: Involving muscle and bone - demonstrated during surgical exploration and either positive cultures or requiring treatment with antibiotics

Prolonged ventilation: Requiring intubation for more than 24 hours

Patients were divided into two groups on the basis of whether the left internal mammary artery (LIMA) was used (group 1) or not used as a conduit (group 2). The two groups were further analyzed in the case of elective and urgent procedures.

Statistical analysis

Data were presented as mean with standard deviation, and discrete variables were presented as frequencies and percentages. For numerical data, the independent sample t-test was applied. For categorical data, the chi-square test was performed. The short-term outcomes analyzed consisted of mortality and major morbidities as per STS. Data entry and analysis were done on IBM SPSS version 22 (IBM Corp., Armonk, NY).

## Results

A total of 2619 patients underwent isolated non-emergent CABG surgery at our institute from January 2010 to December 2016. Of these 2619 patients, 2472 patients received LIMA and 147 patients did not receive LIMA. The 2472 patients who received LIMA were further analyzed on the basis of the nature of the surgery, that is, elective 2117 patients and urgent 355. The 147 patients who did not receive LIMA were also divided into elective (121) and urgent (26) cases. LIMA was used in 94% (n=2472) of patients.

Table 1 demonstrates the differences in preoperative risk variables between the two groups.

**Table 1:** Preoperative clinical characteristics of patients in whom LIMA was used and not used in isolated CABG (n=2619) LIMA: left internal mammary artery; BMI: body mass index; NYHA: New York Heart Association; CPB: cardiopulmonary bypass

Variables	LIMA (n=2472) (94.4%)	No LIMA (n=147) (5.6%)	P
Age in years ± (SD)	59.5±9.7	64.2 ± 11.5	<0.001
Gender			
Male	2039 (82.5)	120 (81.6)	0.792
Women	369 (17.5)	52 (18.3)	
BMI (kg/m^2^)	27±4.4	26.7±5.3	0.388
Diabetes n (%)	1319(53.4)	86 (58.5)	0.224
Hypertension n (%)	1794 (72.6)	111 (75.5)	0.437
Chronic lung disease n (%)	69 (2.8)	6 (4.1)	0.362
Cerebrovascular disease n (%)	79 (3.1)	7 (4.8)	0.186
Preop creatinine (mg/dl) ±SD	1.1±2.3	1.37±1	0.167
Myocardial Infraction	1153 (46.6)	86 (58.5)	0.005
Arrhythmia n (%)	41 (1.7)	6 (4.1)	0.032
NYHA grading n (%)			<0.001
Class I	319 (12.9)	17 (11.6)	
Class II	1224 (49.5)	51(34.7)	
Class III	839 (33.9)	66 (44.9)	
Class IV	90 (3.6)	13 (8.8)	
Diseased vessel n (%)			<0.001
Single	43 (1.7)	8 (5.4)	
Double	328(13.2)	41 (27.8)	
Three	2101 (84.9)	98 (66.6)	
Left main stenosis %	467 (17.6)	41 (21.6)	0.170
Ejection fraction			<0.001
-50%	164 (6.6)	25 (17.0)	
49-30%	757 (30.6)	47 (32.0)	
<30%	1551 (62.7)	75 (51.0)	
Cross-clamp time (min)	65.1±28.3	68.8±30	0.125
CPB time (min)	103±58.2	113±42.5	0.040

Patients undergoing CABG with LIMA were younger, had complex three-vessel coronary artery disease and low ejection fraction. Patients undergoing CABG without LIMA were older, had mostly single or double vessel coronary artery disease, and high NYHA class and preop eGFR. In addition, left main coronary artery disease and acute myocardial infarction were higher in patients having CABG without LIMA but were not statistically significant. Other factors like gender, body mass index (BMI), and major comorbidities, such as diabetes, hypertension, chronic lung disease, and cerebrovascular accident, were comparable between the two groups. There was no statistical difference in the intraoperative cross-clamp and pump time.

Table 2 demonstrates the impact of CABG with and without LIMA used on morbidity and mortality.

**Table 2:** The impact of CABG with and without LIMA used on morbidity and mortality LIMA: left internal mammary artery; CABG: coronary artery bypass grafting; LOS: length of stay

Table 2. Outcome of CABG with or without LIMA			
	LIMA		
Characteristics	Used n=2472	Not Used n=147	P Value*
Major morbidity n (%)	191 (7.7)	31 (21.1)	<0.001
Reopen for any reason n (%)	61 (2.5)	6 (4.1)	0.229
Surgical site infection n (%)	28 (1.1)	4 (2.7)	0.089
Stroke >72 Hrs. n (%)	14 (0.6)	4 (2.7)	0.002
Prolong ventilation n (%)	128 (5.2)	26 (17.7)	<0.001
LOS in Days ± (SD)	6.7±29.2	10.9±10.1	0.078
Mortality	50 (2.0)	13 (8.8)	<0.001

Overall mortality was 63 (2.4%). Patients undergoing CABG with LIMA has significantly lower mortality (2% LIMA vs 8.8% no LIMA) and were less likely to have a major morbidity (7.7% LIMA vs 21.1% no LIMA), prolonged ventilation (5.2% vs 17.7%), and stroke (0.6% vs 2.7%). Other outcomes like surgical site infection, reoperation for any reason, and length of stay (LOS) were not significantly different between the two groups.

Table 3 and Table 4 demonstrate the outcome of CABG with or without the use of LIMA in elective and urgent cases. Patients undergoing elective CABG with LIMA had significantly lower major morbidity (6.8% LIMA vs 15.7% no LIMA) and mortality (1.6% LIMA vs 7.4% no LIMA) as compared to those in whom LIMA was not used. In the case of patients undergoing urgent CABG, LIMA use was associated with significantly lower major morbidity (13% LIMA vs 46.2 % no LIMA); however, mortality (4.8% LIMA vs 15.4% no LIMA) was not significantly different as compared to those in whom LIMA was not used. Table 5 summarizes the overall mortality.

**Table 3:** Demonstrates the outcome of CABG with or without the use of LIMA in elective cases LIMA: left internal mammary artery; CABG: coronary artery bypass grafting

Elective Cases, n=2238			
	LIMA		
Characteristics	Used n=2117	Not Used n=121	P-value*
Major morbidity n (%)	145 (6.8)	19 (15.7)	<0.001
Mortality n (%)	33 (1.6)	9 (7.4)	<0.001

**Table 4:** Demonstrates the outcome of CABG with or without the use of LIMA in urgent cases LIMA: left internal mammary artery; CABG: coronary artery bypass grafting

Urgent Cases, n=381			
	LIMA		
Characteristics	Used n=355	Not Used n=26	P-value*
Major morbidity n (%)	46 (13.0)	12 (46.2)	< 0.001
Mortality n (%)	17 (4.8)	4 (15.4)	0.022

**Table 5:** Mortality by priority of surgery

Characteristics	LIMA	No LIMA	p-value*
Elective	n=2117	n=121	
Major morbidity n (%)	145 (6.8)	19 (15.7)	<0.001
Mortality n (%)	33 (1.6)	9 (7.4)	<0.001
Urgent	n=355	n=26	
Major morbidity n (%)	46 (13)	12 (46.2)	<0.001
Mortality n (%)	17 (4.8)	4 (15.4)	0.022

## Discussion

The LIMA is the gold standard conduit in CABG and has consistently shown to be associated with improved long-term survival, graft patency, and a lower rate of re-intervention as compared with the saphenous vein graft (SVG) conduits [5]. The LIMA has been shown at postmortem examinations to be remarkably free from atherosclerosis [8]. Its superiority is due to its high resistance to atherosclerosis [9-10]; the wall of the IMA is more resistant to atherosclerosis than is the wall of the venous conduit because of differences in muscular layers and in the lamina elastica interna [11]. It is also proved to be a safe conduit in a high proportion of patients with a poor left ventricular ejection fraction [12]. Because of its superior results, the intraoperative usage of at least one (right or left) IMA in CABG is considered as one of the important quality indicators in terms of quality performance measure recommended by the Society of Thoracic Surgeons (STS) [3].

The long-term durability of CABG and a long reoperation free interval has always been the quest for cardiac surgeons since the advent of CABG. Most of the literature is about comparing the long-term outcome of CABG with different conduits [7,13-14]. The LIMA has the highest patency rate of above 90% at 10 years while the saphenous vein graft has a patency rate of 61% at 10 years [15].

There have been fewer studies on the early outcome of CABG with LIMA. Dabal et al. studied the effect of LIMA on early outcomes after CABG, and they found that patients undergoing CABG with LIMA is associated with significantly lower mortality and morbidity [7,16]. However, in their study, most of the patients who did not receive LIMA were older, with a high incidence of renal insufficiency and low ejection fraction. In addition, comorbidities like chronic lung disease and cerebrovascular disease were more common in patients who did not receive LIMA.

Most of the literature on the outcome of CABG with LIMA is on the western population. Fewer studies are performed in South Asia comparing the two groups. The south Asian population has different anatomy of the coronary artery as compared to the western. The left anterior descending artery has a smaller caliber in south Asian as compared to the western, and distal runoff has a major role in the long-term outcome of LIMA. Krishnan et al. from south India compared the outcome in LIMA vs saphenous vein graft and suggested better outcomes in patients undergoing CABG with LIMA. However, their sample size was low and they compared both groups in elective patients [6].

To analyze our data of LIMA used with respect to morbidity or mortality, we found that CABG with LIMA is associated with significantly lower mortality and morbidity in even short-term outcomes. The majority of preoperative and intraoperative variables that included sex, BMI, chronic lung disease, cerebrovascular disease, diabetes, hypertension, and creatinine was comparable in both groups, which was found to be significantly different in other studies [16]. So our study provides a relatively comparable variable for the two groups in terms of major comorbidities. As compared to other studies, in our case, more patients in CABG with LIMA had a complex three-vessel coronary artery disease and low ejection fraction while single or two-vessel disease and moderate reduced ejection fraction were high in the no IMA group. Myocardial infarction and high NYHA classes III and IV were more in the no LIMA group, which was comparable with other studies [7,16]. The result of our study was comparable with the rest of the studies in terms of mortality and major morbidities in terms of prolonged ventilation, stroke, renal dysfunction, surgical site infection, and length of hospital stay [6-7,16].

We also compared the outcome of CABG with and without LIMA in the elective and urgent groups and found that CABG with LIMA is associated with reduced mortality and major morbidity in both the elective and urgent groups. Similarly, female sex is also a commonly referred factor precluding the use of IMA, however, in our study, it was not considered contraindicated despite the fact that they are at high risk of mortality [17]. In the no LIMA group, patients were of older age as compared to the LIMA group and presented with acute myocardial infarction, which was comparable with other studies [6,16].

LIMA is considered as the intraoperative quality of care indicator as per the STS recommendation [3]. To look at whether standard quality measurements are taken in our institute regarding the usage of LIMA, we found that LIMA was used in 94% of the CABG, which was as per the recommendation of the STS quality of care.

The strength of our study is the large sample size, and very few studies have been performed to see the effect of LIMA use with CABG in the South Asian population. And through this study, we found our LIMA usage comparable with the STS recommendation.

The limitation of our study is that we did not study long-term outcomes and the fact that it is a single hospital study. Multiple hospitals in different regions of South Asia should be included for a more uniform result.

## Conclusions

To conclude, LIMA not being used in CABG is associated with significant morbidity and mortality in the short term. And since we used LIMA in more than 90% of the cases, we observe overall significantly lower morbidity and mortality.
